# Characterization of β-adrenergic receptors in bovine intramuscular and subcutaneous adipose tissue: comparison of lubabegron fumarate with β-adrenergic receptor agonists and antagonists

**DOI:** 10.1093/jas/skab116

**Published:** 2021-08-02

**Authors:** Jinhee H Hwang, Michael E Spurlock, John C Kube, Xiang Z Li, Stephen B Smith

**Affiliations:** 1 Department of Animal Science, Texas A&M University, College Station, TX 77843, USA; 2 Nu-Tetics AgVentures, Inc., 12247 CR 8390, West Plains, MO 65775, USA; 3 Elanco Animal Health, 2500 Innovation Way, Greenfield, IN 46140, USA

**Keywords:** Bovine adipose tissue, β-adrenergic receptors, β-adrenergic ligands, cAMP, lipolysis

## Abstract

Chinese hamster ovary cell constructs expressing either the β _1_-, β _2_- or β _3_-adrenergic receptor (AR) were used to determine whether a novel β-AR modulator, lubabegron fumarate (**LUB**; Experior, Elanco Animal Health) might exert greater potency for a specific β-AR subtype. EC_50_ values calculated based on cAMP accumulation in dose response curves indicate that LUB is highly selective for the β _3_-AR subtype, with an EC_50_ of 6 × 10^–9^ M, with no detectible agonistic activity at the β _2_-AR. We hypothesized that the accumulation of lipolytic markers would reflect the agonist activity at each of the β-receptor subtypes of the specific ligand; additionally, there would be differences in receptor subtype expression in subcutaneous (**s.c.**) and intrmuscular (**i.m.**) adipose tissues. Total RNA was extracted from adipose tissue samples and relative mRNA levels for β _1_-, β_2_-, and β _3_-AR were measured using real-time quantitative polymerase chain reaction. Fresh s.c. and i.m. adipose tissue explants were incubated with isoproterenol hydrochloride (**ISO**; β-AR pan-agonist), dobutamine hydrochloride (**DOB**; specific β _1_-AA), salbutamol sulfate (**SAL**; specific β _2_-AA), ractopamine hydrochloride (**RAC**), zilpaterol hydrochloride (**ZIL**), BRL-37344 (specific β _3_-agonist), or LUB for 30 min following preincubation with theophylline (inhibitor of phosphodiesterase). Relative mRNA amounts for β _1_-, β _2_-, and β _3_-AR were greater (*P* < 0.05) in s.c. than in i.m. adipose tissue. The most abundant β-AR mRNA in both adipose tissues was the β _2_-AR (*P* < 0.05), with the β _1_- and β _3_-AR subtypes being minimally expressed in i.m. adipose tissue. ISO, RH, and ZH stimulated the release of glycerol and nonesterified fatty acid (**NEFA**) from s.c. adipose tissue, but these β-AR ligands did not alter concentrations of these lipolytic markers in i.m. adipose tissue. LUB did not affect glycerol or NEFA concentrations in s.c. or i.m. adipose tissue, but attenuated (*P* < 0.05) the accumulation of cAMP mediated by the β _1_- and β _2_-AR ligands DOB and SAL in s.c. adipose tissue. Collectively, these data indicate that bovine i.m. adipose tissue is less responsive than s.c. adipose tissue to β-adrenergic ligands, especially those that are agonists at the β _1_- and β_3_-receptor subtypes. The minimal mRNA expression of the β _1_- and β _3_ subtypes in i.m. adipose tissue likely limits the response potential to agonists for these β-AR subtypes.

## Introduction

For more than 3 decades, the mechanism of action of β-adrenergic agonists (**β-AA**; e.g., cimaterol, clenbuterol, ractopamine, and zilpaterol) has been studied extensively in multiple meat animal species. Depending on species and molecule, β-AA can promote a considerable increase in carcass muscle mass and a reduction in fat accumulation, thus facilitating the production of lean meat as a source of high-quality protein (Dalrymple et al., 1984; Miller et al., 1988; Jones et al., 1985; Moser et al., 1986; Coleman et al., 1988; Schiavetta et al., 1990; Allen et al., 2009; Elam et al., 2009; reviewed in Johnson et al., 2014). Decreased lipid in adipose tissue occurs through the β-adrenergic receptor (**β-AR**)/adenylyl cyclase/cAMP-dependent protein kinase A (**PKA**) signaling cascade which culminates in the activation of perilipin and hormone-sensitive lipase (Wallukat et al., 2002).

Three subtypes of β-AR (β _1_-AR, β _2_-AR, and β _3_-AR) are expressed in tissues of vertebrate species, and the distribution of subtypes and selectivity for synthetic ligands in adipose tissue differs widely among species. For example, the predominant subtype is the β _2_-AR in bovine adipose tissue, whereas β _1_-AR is the primary subtype in porcine adipose tissue (Sillence and Matthews, 1994; McNeel and Mersmann, 1999). The synthetic ligand ICI118,551 is an antagonist for β _2_-AR in both bovine skeletal muscle and adipose tissue, while it has no effect on β _2_-AR in porcine adipose tissue (Sillence and Matthews, 1994; Mersmann, 1998).

Recently, a new synthetic β-AR ligand, lubabegron fumarate (**LUB**, Experior, Elanco Animal Health, Greenfield, IN) has been introduced. This novel β-AR modulator is classified by the Food and Drug Administration’s (**FDA**) Center for Veterinary Medicine (**CVM**) as a β-adrenergic agonist/antagonist. In vitro and in vivo pharmacology studies to assess interaction with multiple receptor systems in humans and comparative animal models indicate that LUF has (1) agonistic activity to the β _3_-AR; (2) high-binding affinity with the β _1_- and β _2_-ARs and low-binding affinity with non- β-ARs; and (3) antagonistic activity to the β _1_- and β _2_-ARs (FDA-FOI, 2018). In this study, we determined the extent to which bovine s.c. and i.m. adipose depots express the β _3_-AR subtype. We also tested the hypothesis that changes in cAMP and lipolytic markers in incubated i.m. and s.c. adipose samples would reflect the β-AR receptor mRNA profile and activation response of each AR subtype.

## Materials and Methods

### Materials

LUB and ractopamine hydrochloride (**RAC**) were provided by Elanco Animal Health (Greenfield, IN), as was zilpaterol hydrochloride (**ZIL**) and a racemic mixture of ractopamine. Other drugs and reagents were purchased from the following companies: isoproterenol hydrocholoride (**ISO**) dobutamine hydrochloride (**DOB**), salbutamol sulfate (**SAL**), ZIL, propranolol hydrochloride (**PRO**), Glycerol Assay Kit and DNAseI (Sigma-Aldrich, St. Louis, MO); L-748,337 and BRL-37344 (R&D Systems, Minneapolis, MN); Cyclic AMP XP Assay Kit (Cell Signaling Technology, Danvers, MA); nonesterified fatty acid (**NEFA**) kit (Wako Life Sciences, Inc., Mountain View, CA); qScript cDNA synthesis kit and Perfecta SYBR Green fastmix (Quanta Biosciences, Gaithersburg, MD).

### Chinese Hamster Ovary cell preparation and identification of active receptor ligands

Chinese Hamster Overy cells were transfected with either the bovine β _1_-, β _2_-, or β _3_-AR constructs in pcDNA3.1+. Expression of the β-AR was confirmed by polymerase chain reaction (**PCR**). Thereafter, cells were grown in DMEM/Ham’s F12 media with 10% FBS and 1% penicillin/streptomycin at 37 °C under 5% CO_2_. Opti-plates (Perkin Elmer, Waltham, MA) were used for all experiments. For each experiment, cells were plated into 96-well tissue culture plates at 60,000 cells per well in a volume of 100 µL media. Cells were grown for 48 hr after plating prior to ligand addition and determination of cAMP.

All samples and standards were tested in duplicate for each repetition, and 4 repetitions per cell line were performed for each potential ligand tested. Controls included no treatment (media alone), 5 mM theophylline alone (an inhibitor of phosphodiesterase), 0.1 % DMSO alone, and water alone. None of the controls differed from media alone and are thus not reported. A volume of media equal to the volume of test material to be added was removed from the well just prior to stimulation. Theophylline was added in a volume of 25 µL to obtain a final concentration of 5 mM and each test molecule was delivered in 10 µL. Cells were returned to 37 °C and allowed to incubate for 45 min before media was removed and the cells lysed in 38 µL lysis buffer A. The LANCE cAMP detection kit (AD0262E) was purchased from Perkin Elmer. The LANCE assay instructions were followed for a 40 µL reaction volume with 15 µL of cell lysate. The reaction was incubated for 20 hr prior to measuring fluorescence. A standard curve was generated with increasing concentrations of cAMP, and the linear portion of the curve was used to calculate sample cAMP concentrations. For LUB, the racemic mix of RAC, and ZIL, cAMP response curves were generated with concentrations increasing from 10^–9^ to 10^–4^ M, and the EC_50_ calculated.

### Animals

The experimental protocols for this study were approved by the Institutional Animal Care and Use Committee at Texas A&M University (College Station, TX), AUP AACUC #2016-009A. Angus cross steers (BW = 498 ± 59 kg, *n* = 20) were fed a standard, corn-based finishing diet at the Texas A&M University McGregor Research Center, McGregor, TX, and then transported ~190 km to the Texas A&M Animal Science Teaching Research and Extension Complex, College Station, TX.

### Bovine adipose tissue sampling

Bovine s.c. and intramuscular i.m. adipose tissues were obtained as described previously (Miller et al., 1988; 1989; 1991; Brooks et al., 2011). Briefly, cattle were stunned using a captive bolt and then exsanguinated. Immediately thereafter, the longissimus muscle (**LM**) and overlying s.c. adipose tissue from the 8th to 10th rib were removed and transported to the laboratory in oxygenated Krebs–Henselheit Ca^2+^-free bicarbonate buffer (**KHB**: 120 mM NaCl, 4.8 mM KCl, 1.2 mM MgSO_4_, 1.2 mM KH_2_PO_4_, 25 mM NaHCO_3_; 37°C; pH 7.4) containing 5 mM glucose and 10 mM HEPES. The elapsed time from stunning to arrival of the muscle at the laboratory was <20 min.

### Adipose tissue explant culture

Fresh s.c. adipose tissue was cut into small pieces (50 to 100 mg, actual weight recorded) and i.m. adipose tissue was dissected from the LM as described previously (Smith and Crouse, 1984; Miller et al., 1988) and weighed. The s.c. and i.m. adipose samples were incubated according to 3 different sets of experimental conditions. Experiment 1: Small pieces of s.c. and i.m. adipose tissues (50 to 100 mg; 1 to 2 mm thick; Smith and Prior, 1981) were preincubated in 6-well tissue culture plates in a CO_2_/O_2_ incubator (NuAire model 4750 CO_2_ incubator, Plymouth, MN) for 30 min at 37 °C, 5% CO_2_ in KHB plus 5 mM glucose, 10 mM HEPES, and 0.5 mM theophylline. After the 30 min preincubation, ISO (β-AR pan-agonist), RAC, ZIL, LUB, and BRL-37344 were added to the culture plate wells to achieve final concentrations of 10^–9^ to 10^–4^ M for an additional 30 min. Experiment 2: The s.c. and i.m. adipose samples were preincubated in KHB plus 5 mM glucose, 10 mM HEPES, and 0.5 mM theophylline in the presence or absence of either 10 µM L-748,337 (β _3_-AR antagonist) or 50 µM PRO (β-AR pan-antagonist). After the 30 min preincubation, LUB was added to the culture wells to achieve final concentrations of 10^–9^ to 10^–4^ M and incubated for an additional 30 min. Experiment 3: The s.c. and i.m. adipose samples were preincubated in KHB plus 5 mM glucose, 10 mM HEPES, and 0.5 mM theophylline in the presence or absence of 1 µM LUB or 1 µM LUB plus 10 µM L-748,337. After the 30 min preincubation, either DOB (β _1_-AA) or SAL (β _2_-AA) was added to the culture plate wells in a concentration-dependent manner (10^–9^ to 10^–4^ M) for an additional 30 min. For all experiments, there were at least 3 replicate wells per treatment condition. After a total of 1-hr incubation, the adipose samples were homogenized in 1 mL cell lysis buffer (Cell Signaling Technology, Danvers, MA) with 1 mM phenymethylsulfony fluoride, and centrifuged at 14,000 × *g* for 30 min to remove tissue debris. The supernatant fractions were stored at −80 °C until subsequent analyses were performed.

### Viability of adipose tissue

Some of the experiments took as much as 60 min before reactions were terminated. Therefore, to confirm viability of adipose samples following incubations with the selected β-AR ligands, lipogenesis was measured in tissue aliquots at the beginning of each experiment (time 0; immediately after samples arrived at the laboratory) and again 60 min later (time 60; to mimic time necessary for the in vitro experiments). Although fatty acid biosynthesis is an enzymatic reaction, the incorporation of acetate requires intact, viable adipose tissue. Even if the adipose tissues lysed completely, substrates such as ATP, CoASH, and NADPH would be diluted at least 30-fold, well below the *K*_m_ for each reaction (Smith and Prior, 1981; Smith and Crouse, 1984). Thus, only intact, viable adipose tissues would incorporate radiolabeled acetate into lipids.

Subcutaneous and i.m. adipose tissue samples (50 to 100 mg) were incubated at time 0 and time 60 as described previously (May et al., 1995). After addition of the adipose tissue to the flasks, flasks were gassed briefly with 95%:5% O_2_:CO_2_ and placed in a shaking water bath for 60 min at 37 °C. Flasks contained oxygenated (95%:5% O_2_:CO_2_), KHB (pH 7.35 to 7.40), 5 mM glucose, 5 mM acetate, 10 mM HEPES, 1 μCi [1-^14^C]acetate (sodium salt, American Radiolabeled Chemicals, Inc., Chicago, IL). After the 60-min incubation, reactions were terminated with the addition of 5% trichloroacetic acid and shaken an additional 30 min. Adipose tissue pieces were removed from the flasks, rinsed with KHB, and placed in tubes containing 10 mL cholorform:methanol (2:1, v/v). Neutral lipids in adipose tissues were extracted (Folch et al., 1957), and total lipids resuspended in 10 mL of scintillation cocktail (Bio-safe2, Research Product International Corp., Mount Prospect, IL). Radioactivity of lipid extracts was determined with a scintillation counter (Packard 1600TR Liquid Scintillation Analyzer, Downers Grove, IL). Results are reported as nanomole acetate converted to fatty acids/(1 hr × 100 mg adipose tissue).

### Cyclic AMP

The concentration of cAMP was determined based on the principle of competitive binding using the Cyclic AMP XP Assay Kit according to the manufacturer’s instructions. Cytosolic extracts from the adipose explants were co-incubated at room temperature for 3 hr in 96-well plates with the HRP-linked cAMP substrate coated onto an immobilized rabbit monoclonal cAMP antibody using a horizontal orbital plate shaker. After the reaction, color development was measured at 450 nm using an Epoch microplate reader (Biotek Instruments, Winooski, VT). All samples were analyzed in duplicate. A standard curve and cAMP concentrations were calculated using GrapPad Prism 6.04 software (GraphPad Software Inc., San Diego, CA).

### Lipolysis

Lipolysis was measured based on changes in glycerol (Sigma-Aldrich, St. Louis, MO) and NEFA (Wako Life Sciences, Inc., Mountain View, CA) concentrations using commercial kits according to the each manufacturer’s protocol. In brief, to determine glycerol released from tissue, 10 µL of the cell extracts were reacted with 100 µL glycerol reaction reagent for 20 min at room temperature and the absorbance read at 570 nm. The glycerol standard provided in the assay kit was used for the calibration curve. To analyze the level of NEFA released from the adipose tissues, 25 µL of cell extract plus 200 μL of the first reagent were incubated for 5 min at 37 °C, followed by the addition of 100 µL of the second reagent and a second 5-min incubation at 37 °C. Thereafter, the absorbance was read at 550 nm. Oleic acid was used to plot a standard curve for calculation of the NEFA concentration.

### Real-time quantitative PCR (RT-qPCR)

Approximately 200 mg of each adipose sample (*n* = 10 per depot) was used to isolate total RNA using a combination of the Trizol reagent (Invitrogen, Carlsbad, CA) and the HiBind RNA mini column (Omega Bio-Tek, Inc., Norcross, GA). After treatment with DNAseI, total RNA was quantified on a NanoDrop 2000 Spectrophotometer (Thermo Fisher Scientific, Waltham, MA) and reverse transcription was performed with the qScript cDNA synthesis kit. The mRNA expression of the β-AR subtypes in s.c. and i.m. adipose samples was analyzed in a CFX384^TM^ Real-Time System (Bio-Rad, Hercules, CA) using the Perfecta SYBR Green fastmix kit. The different efficacy of cDNA synthesis between samples was normalized with 3 reference genes (ribosomal protein 9, *RSP9*; glyceraldehyde-3-phosphate dehydrogenase, *GAPDH*; and succinate dehydrogenase, *SDHA*). The relative expression of mRNA was determined by the cycle threshold (**CT**) deviation of an unknown sample vs. geometric mean of the three reference genes. The data are presented as 2^−ΔCT^. The primers used in this assay are listed in Table 1.

**Table 1.** Primers for RT-qPCR

Gene	Accession number	Sequence	Amplicon length (bp)
ADRB1	NM_194266.1	F: 5′-CAGAAGGCACTCAAGACGCT-3′	81
		R: 5′-CACCACGTTGGCTAGGAAGA-3′	
ADRB2	NM_174231.1	F: 5′-TGATCGCTGTGGATCGCTAC-3′	149
		R: 5′-CCGGTACCAGTGCATCTGAA-3′	
ADRB3	NM_174232.2	F: 5′-ACCTTCATTCTGTTCCTTCTG-3′	145
		R: 5′-CTGTGAGGTAGGTGTGTCTA-3′	
GAPDH	NM_001034034.2	F: 5′-CTGCCCGTTCGACAGATAG-3′	76
		R: 5′-CTCCGACCTTCACCATCTTG-3′	
RPS9	NM_001101152.2	F: 5′-GAGCTGGGTTTGTCGCAAAA-3′	65
		R: 5′-GGTCGAGGCGGGACTTCT-3′	
SDHA	NM_174178.2	F: 5′-ACCTGATGCTTTGTGCTCTG-3′	106
		R: 5′-TCGTACTCGTCAACCCTCTC-3′	

### Statistical analysis

The data are expressed as mean ± SEM. Statistical analysis of the change in gene expression obtained by RT-qPCR was tested with a 2-sided, unpaired student’s *t*-test and Tukey’s honest significant difference test using JMP Pro 12 software (SAS Institute Inc., Cary, NC). A *P*-value of <0.05 was considered significant. Nonlinear regression was used to determine the effects of test molecules on the lipolytic response. One-way analysis of variance was used where it was appropriate for statistical comparison of molecule effects with the control and different β-AR ligands using GraphPad prism 6.0.

## Results

### Induction of cAMP in CHO cells specific for bovine β _1_-, β _2_-, or β _3_-AR expression

Each of the ligands tested effectively induced cAMP in the CHO cell assay system and showed selectivity for specific receptors (Table 2). ZIL had greatest activity (lowest EC_50_) with the β _2_ receptor and no detectible cAMP response when incubated with cells expressing the β _1_ receptor. LUB was highly selective for the β _3_ receptor, with no detectible activity in the cells expressing β _2_, and considerably less potency in the β _1_ cell system. In contrast, the racemic mix of RAC showed its greatest potency in cells expressing β _1_ but was also effective in cells expressing either β _2_- or β _3_-AR.

**Table 2.** Induction of cAMP (EC_50_, M) in CHO cells transfected with bovine β _1_-, β _2_-, or β _3_-adregernic receptor constructs^1^

Treatment	β _1_-Construct	β _2_-Construct	β _3_-Construct
ZIL	ND^2^	8.9 × 10^–8^	9.1 × 10^–6^
RAC^3^	2.0 × 10^–9^	4.6 × 10^–8^	2.4 × 10^–8^
LUB	1.9 × 10^–7^	ND^1^	6.0 × 10^–9^

^1^The concentration of cAMP was determined using the LANCE cAMP detection kit (AD0262E) according to the manufacturer’s instructions. Cell lysates were incubated for 20 hr prior to measuring fluorescence. A standard curve was generated with increasing concentrations of cAMP, and the linear portion of the curve was used to calculate sample cAMP concentrations. A standard curve was generated with increasing concentrations of cAMP, and the linear portion of the curve was used to calculate sample cAMP concentrations.

^2^ND indicates that cAMP accumulation was either not detected or insufficient to allow a response curve EC_50_ calculation.

^3^Racemic mixture of RAC.

### β-AR mRNA expression in s.c. and i.m. adipose tissue

**Figure 1.**
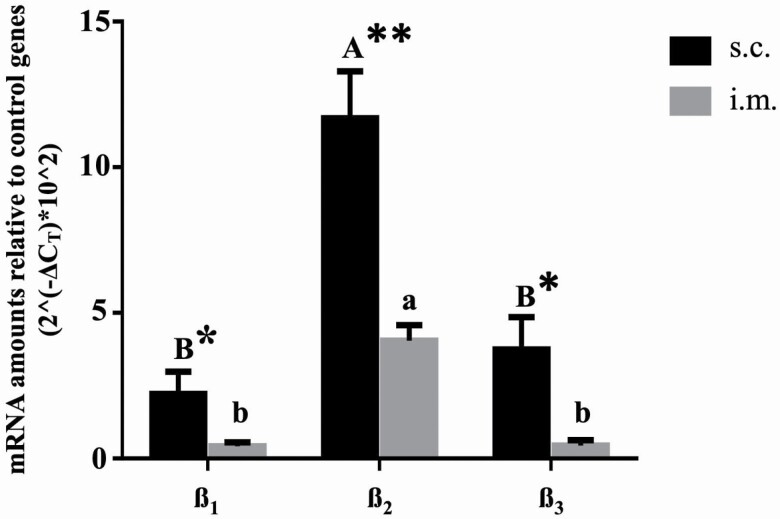
β-Adrenergic receptor gene populations in growing steers analyzed by RT-qPCR. The different efficacy of cDNA synthesis between samples was normalized with 3 reference genes (ribosomal protein 9, *RSP9*; glyceraldehyde-3-phosphate dehydrogenase, *GAPDH*; and succinate dehydrogenase, *SDHA*). The relative expression of mRNA was determined by the CT deviation of an unknown sample vs. geometric mean of the three reference genes. Data are expressed as means ± SEM (*n* = 10). ^AB^Means within s.c. adipose tissue not sharing common superscripts differ (*P* < 0.05). ^ab^Means within i.m. adipose tissue not sharing common superscripts differ (*P* < 0.05). * *P* < 0.05, **< *P* < 0.001 s.c. vs. i.m. adipose tissue.

Expression of all 3 β-AR subtypes was detected in bovine s.c. and i.m. adipose depots (Figure 1), with expression of the β _2_-AR being greater (*P* < 0.05) than that of either the β _1_- or the β _3_-AR subtype, irrespective of depot. The expression of the β _3_-AR was not different from β _1_-AR expression (*P* > 0.05) in either adipose depot. The β-AR subtypes were 5.3, 2.9, and 8.3 times higher in s.c. than i.m. adipose tissue for the β _1_-, β _2_-, and β _3_-AR, respectively (*P* < 0.05), and the expression of the β _2_-AR in s.c. adipose tissue was 5.3 and 3.1 times higher than that of the β _1_- and β _3_-AR, respectively. The β _2_-AR mRNA level was 9.6 and 10 times greater than expression of the β _1_- and β _3_-AR, respectively, in i.m. adipose tissue.

### Lipogenesis

Fatty acid biosynthesis from acetate was greater (*P* < 0.05) in s.c. adipose tissue 60 min after sample arrival at the laboratory (time 60) than rates observed in samples immediately upon arrival at the laboratory (time 0; Table 3). Fatty acid biosynthesis from acetate did not differ (*P* > 0.05) between time 60 and time 0 for i.m. adipose tissue. We conclude that there was no indication of loss of viability during the preincubation/incubation period.

**Table 3.** Fatty acid synthesis from acetate in subcutaneous and intramuscular adipose tissue incubated time 0 (immediately after samples arrived at the laboratory) and time 60 (60 min after samples arrived at the laboratory)^1^

Incubation time, min	Time 0	Time 60
s.c.	45.56 ± 8.57^b^	89.63 ± 17.79^a^
i.m.	26.65 ± 8.46^a^	39.21 ± 9.91^a^

^1^Values are mean ± SEM for *n* = 16 steers. Rates are nanomole acetate converted to fatty acids per 100 mg adipose tissue per 1 hr incubation. ^ab^Means within a row with common superscripts do not differ (*P* > 0.05). The main effect of tissue (subcutaneous vs. intramuscular) was significant (*P* = 0.003).

### Accumulation of cAMP in s.c. and i.m. adipose tissue in response to β-AR ligands

**Figure 2.**
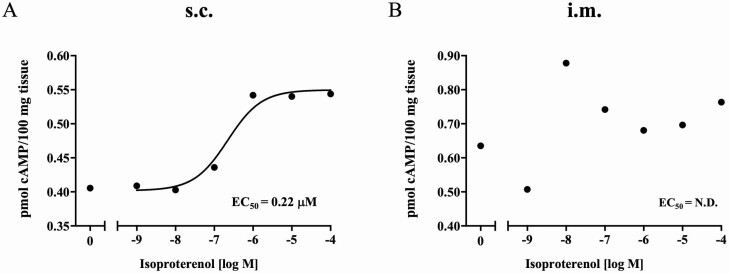
cAMP production in s.c. (A) and i.m. (B) adipose tissue in response to ISO hydrochloride (IH). Tissues were incubated with IH for 30 min at 37 °C. Data are expressed as mean values (*n* = 5). The results at each concentration of IH were fitted by nonlinear regression. The concentration of cAMP was determined using the Cyclic AMP XP Assay Kit according to the manufacturer’s instructions, as described in Table 2.

The β-AR pan-agonist, ISO, the β _1_-and β _2_-AA RH, and the β _2_-AA ZIL were used to assess the cAMP signaling cascade in s.c. and i.m. adipose tissue. ISO hydrochloride increased cAMP accumulation in s.c. adipose tissue, with the response plateauing at 10^–6^ M (Figure 2A). The calculated EC_50_ for ISO in s.c. adipose tissue was 0.22 µM. In i.m. adipose tissue, the concentration of cAMP was slightly higher than in s.c. adipose tissue (Figure 2B). However, cAMP accumulation was not dose-dependent for ISO in i.m. adipose tissue. Neither RAC nor ZIL (10^–8^ to 10^–6^ M) affected cAMP concentrations (*P* > 0.05) in s.c. or i.m. adipose tissue (Table 4).

**Table 4.** Concentrations of cAMP, glycerol, and NEFA in extracts of subcutaneous (s.c.) and intramuscular (i.m.) adipose tissue incubated with RAC or ZIL^1^

	RAC		ZIL	
	s.c.	i.m.	s.c.	i.m.
*cAMP (pmol/100 mg tissue)* ^*2*^				
Baseline	0.42 ± 0.06	0.64 ± 0.19	0.42 ± 0.06	0.64 ± 0.19
10^–8^	0.39 ± 0.09	0.86 ± 0.22	0.76 ± 0.37	0.79 ± 0.30
10^–7^	0.38 ± 0.08	0.76 ± 0.18	0.61 ± 0.27	0.51 ± 0.13
10^–6^	0.44 ± 0.14	0.70 ± 0.18	0.62 ± 0.27	0.78 ± 0.39
*Glycerol (nmol/100 mg tissue)*				
Baseline	50.0 ± 12.8	48.6 ± 10.3	50.0 ± 12.8	48.6 ± 10.3
10^–8^	50.3 ± 7.60	52.4 ± 16.4	54.8 ± 8.0	67.4 ± 21.6
10^–7^	52.6 ± 11.1	81.7 ± 25.5	59.3 ± 15.4	48.6 ± 13.7
10^–6^	63.3 ± 9.20	42.6 ± 11.4	65.9 ± 12.6	46.0 ± 14.5
*NEFA (µmol/100 mg tissue)*				
Baseline	0.99 ± 0.40	0.65 ± 0.20	0.99 ± 0.40	0.65 ± 0.20
10^–8^	1.10 ± 0.15	0.75 ± 0.21	1.06 ± 0.30	0.78 ± 0.28
10^–7^	1.20 ± 0.21	1.18 ± 0.50	1.25 ± 0.31	0.91 ± 0.30
10^–6^	1.06 ± 0.13	0.56 ± 0.13	1.17 ± 0.35	0.50 ± 0.16

^1^The concentration of cAMP was determined using the Cyclic AMP XP Assay Kit according to the manufacturer’s instructions, as described in Table 2. Lipolysis was measured based on changes in glycerol and NEFA concentrations using commercial kits according to the each manufacturer’s protocol. To determine glycerol released from tissue, cell extracts were reacted with glycerol reaction reagent and the absorbance read at 570 nm. The glycerol standard provided in the assay kit was used for the calibration curve. To determine NEFA released from the adipose tissues, cell extract plus first reagent were incubated, followed by the addition of the second reagent. The absorbance was read at 550 nm. Oleic acid was used to establish a standard curve for calculation of the NEFA concentration.

^2^Values are mean ± SEM; *n* = 6 steers. Means within treatment and tissue were not different (*P* > 0.05).

### ISO hydrochloride, RAC, and ZIL-stimulated lipolysis in s.c. and i.m. adipose tissue

**Figure 3.**
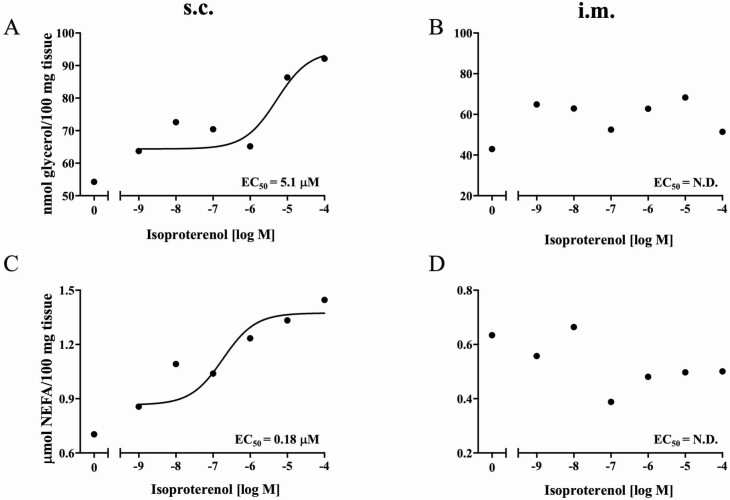
Glycerol and NEFA release in s.c. (A and C) and i.m. (B and D) adipose tissue in response to ISO hydrochloride (IH). Tissues were incubated with IH for 30 min at 37 °C (*n* = 5). Glycerol (A and B) and NEFA (C and D) released in tissue supernatant were determined. Data are expressed as mean values (*n* = 5). Lipolysis was measured based on changes in glycerol and NEFA concentrations using commercial kits as described in Table 4. The results at each concentration of IH were fitted by nonlinear regression.

To determine the effects ISO, RAC, and ZIL on lipolysis, the release of glycerol and NEFA from s.c. and i.m. adipose explants was measured. ISO hydrochloride increased glycerol release in a concentration-dependent manner in s.c. adipose tissue (EC_50_ = 5.1 µM), but had no effect in i.m. explants (Figure 3A and B). Neither RAC nor ZIL affected cAMP production or glycerol or NEFA release in s.c. or i.m. adipose tissue (Table 4). ISO hydrochloride increased NEFA release in a dose-dependent manner in s.c. adipose tissue (EC_50_ = 0.18 µM) (Figure 3C). However, ISO failed to elicit a similar response curve in i.m. explants (Figure 3D).

### Lipolytic response to BRL-37344 and LUB for β _3_-AR

**Figure 4.**
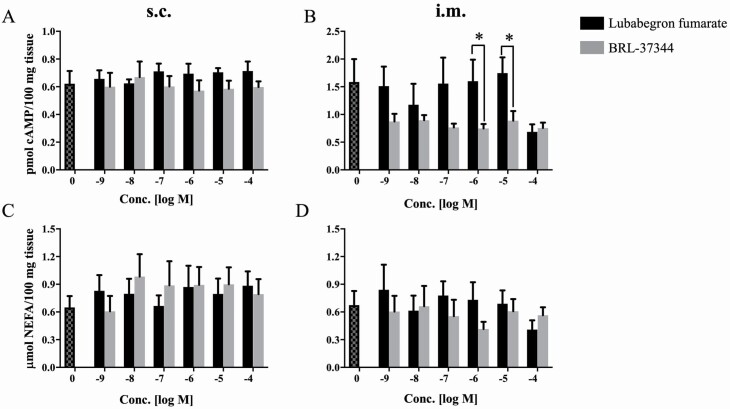
cAMP and NEFA release in response to BRL-37344 and LUB in s.c. (A and C) and i.m. (B and D) adipose tissue. Tissues were incubated with either BRL-37344 or LUB for 30 min at 37 °C. Data are expressed as mean ± SEM (*n* = 6). *P < 0.05 LUB vs. BRL-37344. The concentration of cAMP was determined using the Cyclic AMP XP Assay Kit according to the manufacturer’s instructions, as described in Table 2. Lipolysis was measured based on changes in glycerol and NEFA concentrations using commercial kits as described in Table 4.

To determine if binding of LUB to β _3_-AR might elicite a lipolytic response, the β _3_-AA BRL-37344 (a potent β _3_AR agonist with high affinity for the bovine β _3_-AR; Piétri-Rouxel et al., 1995) was tested for comparison. The EC_50_ value for LUB was similar to that of BRL-37344 in s.c. adipose tissue, whereas a response curve with a calculable EC_50_ for LUB was not demonstrable in i.m. adipose tissue (Table 5). Neither BRL-37344 nor LUB influenced cAMP or NEFA concentrations in s.c. adipose tissue (Figure 4A and C). The concentration of cAMP in i.m. adipose tissue treated with 10^–6^ and 10^–5^ M BRL-37344 was significantly less (*P* < 0.05) than in explants treated with the same concentrations of LUB, but NEFA release was not influenced in i.m. adipose tissue by either ligand (Figure 4B and D).

**Table 5.** Adipose tissue lipolytic sensitivity (−logEC_50_) to β-AR ligands^1^

	s.c.	i.m.
LUB	7.40 ± 1.78^2^	N.A.^3^
BRL-37344	7.00 ± 3.27	N.A.
SAL	6.23 ± 0.35	4.59 ± 4.42
SAL + LUB	3.34 ± 0.59 ***	8.07 ± 4.90
SAL+ LUB + L-748,337	4.04 ± 0.34 ***	N.A.
DOB	5.50 ± 0.68	6.52 ± 1.23
DOB + LUB	6.44 ± 3.20	7.29 ± 1.44
DOB+ LUB + L-748,337	5.87 ± 0.81	6.12 ± 1.70

^1^The concentration of cAMP was determined using the Cyclic AMP XP Assay Kit according to the manufacturer’s instructions, as described in Table 2.

^2^Values are mean ± SEM of sensitivity of adipose tissue (−logEC_50_); *n* = 12 cattle. EC_50_ was calculated from the individual concentration-response to agonists fitted together by nonlinear regression. EC_50_, half maximum effective response.

^3^NA, not applicable. It was not possible to calculate EC_50_ from the data.

***P < 0.001, vs. SAL.

### General β-AR antagonism by β-AR antagonists

**Figure 5.**
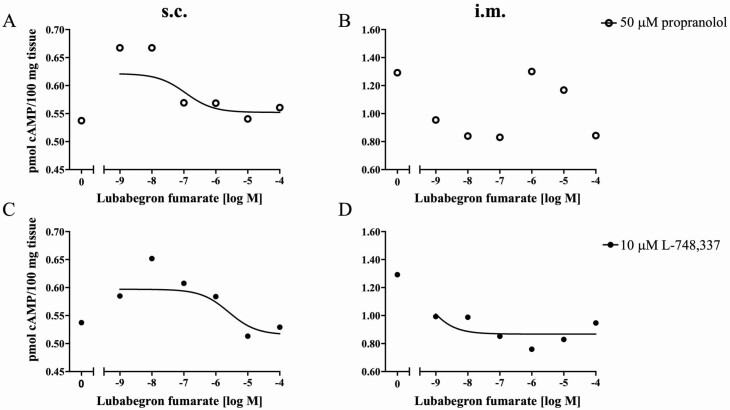
Depression of cAMP production in response to propranolol hydrochloride (PH) (A and B) and L-748,337 (C and D) in s.c. and i.m. adipose tissue. Tissues were preincubated with 10 µM L-748,337 or 50 µM propranolol, and then LUB was added for an additional 30 min. The concentration of cAMP was determined using the Cyclic AMP XP7 Assay Kit according to the manufacturer’s instructions, as described in Table 2. Data are expressed as mean values (*n* = 8). The results at each concentration of LUB were fitted by nonlinear regression.

We set the concentrations of PRO, a β-AR pan-antagonist at 50 µM, and L-748,337, a β _3_-AR antagonist at 10 µM based on previous reports (Hall and Saggerson, 1985; Candelore et al., 1999). A recent report confirmed the efficacy of 10 µM L-748,337 in 3T3-L1 adipocytes (Chen et al., 2021). Subcutaneous and i.m. adipose tissues were preincubated with either 50 µM PRO or 10 µM L-748,337 for 30 min before adding LUB in increasing concentrations. The production of cAMP was reduced by LUB in a dose–response manner in s.c. adipose tissue pretreated with either 50 µM PRO or 10 µM L-748,337 (Figure 5A and C). There was no effect of LUB on cAMP production in i.m. adipose tissue preincubated with PRO (Figure 5B). However, LUB depressed cAMP production in i.m. adipose tissue preincubated with L-748,337 (Figure 5D).

### Antagonistic effects of LUB on β _1_- and β _2_-AR

**Figure 6.**
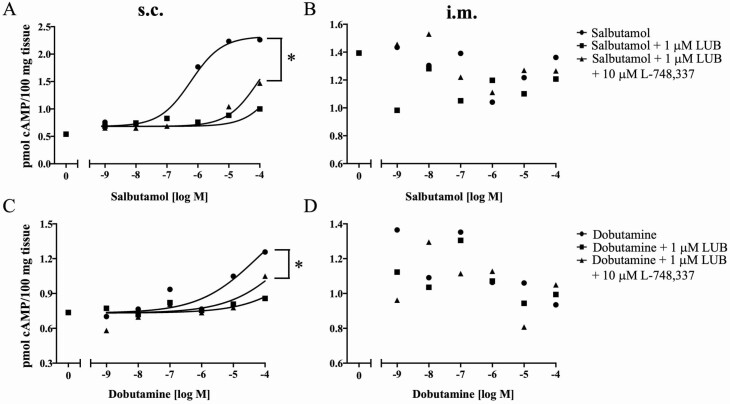
cAMP production in response to β _1_- and β _2_-AR agonists in s.c. (A and C) and i.m. adipose tissue (B and D). Tissues were preincubated with 1 µM LUB or 1 µM LUB + 10 µM L-748,337, and then salbutamol (A, B) or dobutamine (B, D) was added for an additional 30 min. The concentration of cAMP was determined using the Cyclic AMP XP Assay Kit according to the manufacturer’s instructions, as described in Table 2. The results of each individual concentration-response to salbutamol and dobutamine were fitted by nonlinear regression. Data are means (*n* = 5). *P < 0.05.

In s.c. adipose tissue, LUB blunted the production of cAMP in the presence of β-AR antagonists, suggesting that LUB also is a β-AR antagonist (Figure 5). To address this hypothesis, a β _1_-AA (DOB) and a β _2_-AA (SAL) were used to stimulate cAMP production predominantly by the targeted receptor subtypes. Dose–response curves for cAMP production induced by either DOB or SAL in s.c. and i.m. adipose tissue are indicated in Figure 6, and EC_50_ values are summarized in Table 5. Both SAL and DOB increased cAMP production in s.c. adipose tissue in a dose-dependent manner (Figure 6A and C), but were without effect in i.m. adipose tissue (Figure 6B and D). To investigate the antagonism of LUB at the β _1_- and β _2_-AR, s.c. and i.m. adipose tissues were pretreated with 1 µM LUB in the presence or absence of 10 µM L-748,337 before adding DOB or SAL. LUB alone or in combination with 10 µM L-748,337 significantly increased (*P* < 0.0001) the EC_50_ of the SAL cAMP response in s.c. adipose tissue (Figure 6A) and attenuated the maximal cAMP response (*P* < 0.05). There was not a significant change in the EC_50_ of the cAMP response to DOB attributable to LUB alone or in combination with L-748,337 in s.c. adipose tissue (*P* > 0.05). However, LUB, alone or in combination with L-748,337, significantly diminished the maximal cAMP response to DOB in s.c. adipose tissue (*P* < 0.05; Figure 6C). Neither DOB nor SAL altered cAMP concentrations in i.m. adipose tissue (Figure 6B and D). Likewise, LUB was without effect in the i.m. adipose explants.

## Discussion

β-AR are G protein-coupled receptors, and 3 subtypes (β _1_-AR, β _2_-AR, and β _3_-AR) have been identified in mammalian cells. The U.S. FDA recently approved LUB (type A medicated article) for use in beef cattle to reduce the release of ammonia gas from the animal and its waste. Because LUB elicits either agonistic or antagonistic properties as regards specific β-AR subtypes, we sought to establish a more detailed understanding of the regulation of cAMP by this novel β-AR ligand in a CHO cell system in which specific bovine β-AR subtypes were expressed, and in bovine adipose explants prepared from s.c. and i.m. adipose tissue. We used cAMP, glycerol, and NEFA concentrations as indicators of the adenylyl cyclase/PKA/hormone sensitive lipase lipolytic cascade. Our findings indicate several important aspects of LUB and the β-AR populations in bovine adipose tissue.

Results obtained in our CHO cell system indicate that whereas LUB stimulated cAMP accumulation through both the β _1_- and β _3_-AR subtypes, it exerted much greater potency (lower EC_50_) at the β _3_-AR, and did not exert detectible agonistic activity at the β _2_-AR. This selectivity for the β _3_-AR was quite unique vs. ZIL and the racemic mixture of RAC, and may improve our ability to manipulate beneficial effects mediated via the β _3_-AR, while minimizing undesirable outcomes resulting from agonistic activity at the β _1_- or β _2_-AR.

We detected mRNAs for each of the 3 subtypes (β _1_, β _2_, and β _3_), with β _2_ being most highly expressed in both i.m. and s.c. adipose tissue, and with expression of all subtypes being higher in s.c. than i.m. adipose tissue. The gene expression of the β _3_-AR subtype was shown previously to be expressed in brown adipose tissue in cattle, s.c. adipose tissue in pigs, most tissues of sheep, and s.c. adipose tissue, mammary gland, gastrointestinal tracts, and liver of dairy cattle (Casteilla et al., 1994; Piétri-Rouxel et al., 1995; McNeel and Mersmann, 1999; Inderwies et al., 2003; Meylan et al., 2004; Carron et al., 2005; Kobel et al., 2006; Sumner and McNamara, 2007; Wu et al., 2011). Although we cannot extrapolate mRNA data to receptor protein content, this is the first report confirming the mRNA expression of the β _3_-AR (i.e., *ADRB3*) in s.c. and i.m. adipose tissues of beef cattle, and showing adipose depot- and subtype-specific differences in β-AR mRNA abundance. This indicates that each β-AR subtype may be transcriptionally regulated to vary the proportional relationship of receptor subtypes on the cell surface, and also indicates the potential to manipulate specific pathways via specific β-AR subtype activation.

We recently demonstrated that treatment of beef steers depressed i.m. adipocyte volume (Gonzales et al., 2020). Taken together with the high affinity of β _2_-AR subtype for ZH in CHO cells and the greater abundance of β _2_-AR mRNA than the other β-AR subtypes in i.m. adipose tissue, we can conclude the ZH depresses bovine i.m. adipocyte volume via the β _2_-AR. In contrast, the β _2_-AR has no affinity for LUB and s.c. and i.m. adipose tissue exhibited low expression of β _3_-AR. We predict that i.m. adipocyte size, hence marbling scores, would not be affected in LUB-treated cattle.

Of particular interest in the present study was the determination of the extent to which LUB regulates cAMP concentrations and lipolysis. First of all, our experiments show that LUB, which is an agonist at the β _3_-AR, does not invoke a concentration-dependent increase in either cAMP or NEFA in s.c. or i.m. explants. We interpret these findings to indicate that LUB is not a potent β-AR agonist in bovine s.c. or i.m. adipose explants. Although the mechanistic reason for this lack of response is not apparent from our data, it should be noted that BRL-37344 (a classical β _3_-agonist) also failed to alter cAMP and NEFA concentrations. It is possible that this reflects the low β _3_ mRNA abundance, particularly in i.m. adipose tissue, or that the β _3_-AR is not tightly coupled to lipolysis in bovine s.c. and i.m. adipose tissue. Similar results were reported previously in porcine adipose tissue by Mills (2000) in that BRL-37344 did not increase cAMP production or increase lipolysis. Although the β _3_-AR is largely responsible for the lipolytic response to β _3_-AA such as CL-316243 and BRL-37344 in rodents, rabbits, and dogs, it is poorly responsive to β _3_-AA in humans, primates, guinea pigs, and pigs (Bousquet-Mélou et al., 1994; Carpéné et al., 1994; Langin et al., 1995; Mills, 2000). Our findings underscore the question as to the physiological function of the β _3_-AR in bovine adipose tissue.

Also of considerable interest was the finding that LUB antagonized the induction of cAMP in s.c. explants by DOB and SAL (β _1_ and β _2_ agonists, respectively). This antagonism encompassed an increased EC_50_ and reduced maximal response. Because cardiac and bronchial airway responses to β-AA are largely mediated by the β _1_- and β _2_-AR subtypes, respectively, the identification of a molecule that is an agonist at the β _3_-AR, while also acting as an antagonist at the β _1_-and β _2_-AR, may offer unique opportunities in meat animals.

ISO, RAC, and ZIL stimulated glycerol and NEFA release from s.c. adipose tissue. In contrast, those β-AA were not effective in elevating cAMP production or the release of glycerol or NEFA in i.m. adipose tissue. LUB significantly inhibited stimulation of cAMP production mediated by interaction of β _1_- and β _2_-AR and adenylyl cyclase in s.c. adipose tissue, whereas LUB did not have any effect in i.m. adipose tissue.

β-Adrenergic agonists act as repartitioning agents that lead to the redirection of nutrients from lipid synthesis to protein synthesis, modulating animal growth in various species, including cattle, pigs, poultry, and birds (Jones et al., 1985; van Weerdon, 1987; Moloney et al., 1990; Schiavetta et al., 1990; Smith et al., 1995). Oral administration of these β-AA (e.g., cimaterol, clenbuterol, L-644,969, and RAC) increased muscle mass by increasing the ratio of protein to DNA, elevating myofibrillar protein synthesis (Smith et al., 1989, 1995), and depressing myofibrillar protein degradation (Wang and Beermann, 1988), whereas β-AA depress adipose tissue accretion in livestock species by directly stimulating triacylglycerol degradation and by inhibiting fatty acid and triacylglycerol synthesis. The effects of β-AA begin with the stimulation of β-AR through the G-coupled proteins which activate adenylyl cyclase which, in turn, stimulates production of cAMP. In the current study, ISO was chosen as the non-specific agonist for all β-AR subtypes, to compare the effects of ZIL and RAC on cAMP-dependent lipolysis in response to β-AR stimulation. After 30 min incubation, ISO increased cAMP production and, consequently, induced increases in glycerol and NEFA release from s.c. adipose tissue in dose-dependent manner.

Neither ZIL nor RAC affected cAMP production or the release of glycerol or NEFA in i.m. or s.c. adipose tissue. Activation of β-AR stimulated by β-AA, including ISO, cimaterol, clenbuterol, and RAC, in porcine adipose tissue increased the release of glycerol and NEFA (Peterla and Scanes, 1990). Moreover, oral administration of RAC to pigs reduced fat accretion owing to suppression of the activity of lipogenic enzymes and, in turn, the depression of de novo fatty acid synthesis (Mills et al., 1990). Additionally, gene expression associated with lipid synthesis including *sterol regulatory element binding protein-1*, *fatty acid synthase*, and *proliferator-activated receptor-γ2* also was reduced in pigs by RAC (Reiter et al., 2007; Halsey et al., 2011). Page et al. (2004) postulated that RAC can trigger apoptosis in mouse adipose tissue. Miller et al. (1988) demonstrated that treatment of heifers with clenbuterol depressed s.c. and i.m. adipocyte volume, corresponding to decreased activities associated with de novo fatty acid biosynthesis (e.g., fatty acid synthase and NADP-malate dehydrogenase). Taken together, reduction in fat accretion induced by β-AA may be through increased lipolysis and depressed lipogenesis.

Individual β _1_-AR or β _2_-AR subtypes have different abilities to elicit cAMP production. DOB, a β _1_-AA, modestly increased cAMP production, while SS, a β _2_-AA, strongly evoked cAMP production in s.c. adipose tissue, suggesting that β _2_-AR is the primary regulator of lipolysis in cattle, and that the β _1_-AR has a lesser function in lipolysis.

To investigate the antagonism of LUB, we co-incubated LUB with tissues pretreated with PRO, a β-AR pan-antagonist. The production of cAMP was depressed strongly by increasing LUB concentrations. Further, following pretreatment with LUB, LUB attenuated the ability for SAL and DOB to increase cAMP accumulation. This supports the concept that LUB functions better as an antagonist for β _1_-AR or β _2_-AR than as a β _3_-AA in bovine adipose tissue. Baker et al. (2003) proposed the existence of 2 separate binding sites of the human β _1_-AR: (1) 1 for classic agonists and β-antagonists and (2) the other for another agonist (i.e., CGP 12177). CGP 12177 is an agonist that is relatively resistant to inhibition by PRO and CGP 20712A (Konkar et al., 2000). The results of the current study demonstrated that DOB and SAL had agonistic effects on individual β _1_-AR and β _2_-AR, respectively, and also depressed cAMP production by LUB at higher concentrations of DOB and SAL. Furthermore, the combination of PRO plus LUB decreased cAMP production. This suggests that bovine β _1_-AR or β _2_-AR may have 2 separate binding sites, and each β-AA and LUB may act on different binding sites in bovine adipose tissue.

In the current study, i.m. adipose tissue did not show reproducible lipolytic responses to β-AA, although cAMP production was greater in i.m. adipose tissue than in s.c. adipose tissue for all experiments. Adipocyte cell diameter and volume in i.m. adipose tissue are less than in s.c. adipose tissue (Smith and Crouse, 1984; Miller et al., 1989). Subcutaneous adipose tissue may develop initially as brown adipose tissue, subsequently dedifferentiating and redifferentiating to white adipose tissue (Landis et al., 2002). The current study demonstrated that the levels of gene expression of β-AR were much lower in i.m. adipose tissue than that of s.c. adipose tissue, which suggests lesser amounts of β-AR populations in i.m. adipose tissue. Therefore, i.m. adipose tissue apparently would be less responsive to lipolysis induced by synthetic or parasympathetic stimulation than s.c. adipose tissue.

In conclusion, our results indicate the potential specificity of s.c. and i.m. adipose tissues in the expression of particular β-AR subtypes during cattle growth, as well as different physiological responses of β-AR subtypes to β-AA administration. We investigated cAMP accumulation and lipolysis mediated by the interaction of β-AR and β-AA, in which s.c. adipose tissue explants were more responsive to β-AA treatment. LUB, a novel β-modulator which is classified by the CVM as a β-adrenergic agonist/antagonist, is both a β _3_-AR agonist and a β _1_-and β _2_-AR antagonist. These unique combinations of agonistic and antagonistic effects may have confounding impacts on lipolysis and muscle hypertrophy compared to traditional β-AA supplementation to cattle.

## Abbreviations

ATP: adesosine triphosphate
cAMP: cyclic adenosine monophosphate
cDNA: complementary deoxyribonucleic acid
CoASH: coenzyme A
DOB: dobutamine hydrochloride
ISO: isoproterenol hydrocholoride
LM: longissimus dorsi muscle
LUB: lubabegron fumarate
mRNA: messenger ribonucleic acid
NAPDH: nicotinamide dinucleotide reduced
NEFA: nonesterified fatty acid
PCR: polymerase chain reaction
PKA: protein kinase A
PRO: propranolol hydrochloride
RAC: ractompamine hydrochloride
RNA: ribonucleic acid
RT-qPCR: real-time quantitative polymerase chain reaction
SAL: salbutamol sulfate
β-AA: β-adrenergic agonist
ß-AR: β-adrenergic receptor
ZIL: zilpaterol hydrochloride
